# Gene–Environment Interaction in Yeast Gene Expression

**DOI:** 10.1371/journal.pbio.0060083

**Published:** 2008-04-15

**Authors:** Erin N Smith, Leonid Kruglyak

**Affiliations:** 1 Lewis-Sigler Institute for Integrative Genomics and Department of Ecology and Evolutionary Biology, Princeton University, Princeton, New Jersey, United States of America; 2 Molecular and Cellular Biology Program, University of Washington, Seattle, Washington, United States of America; North Carolina State University, United States of America

## Abstract

The effects of genetic variants on phenotypic traits often depend on environmental and physiological conditions, but such gene–environment interactions are poorly understood. Recently developed approaches that treat transcript abundances of thousands of genes as quantitative traits offer the opportunity to broadly characterize the architecture of gene–environment interactions. We examined the genetic and molecular basis of variation in gene expression between two yeast strains (BY and RM) grown in two different conditions (glucose and ethanol as carbon sources). We observed that most transcripts vary by strain and condition, with 2,996, 3,448, and 2,037 transcripts showing significant strain, condition, and strain–condition interaction effects, respectively. We expression profiled over 100 segregants derived from a cross between BY and RM in both growth conditions, and identified 1,555 linkages for 1,382 transcripts that show significant gene–environment interaction. At the locus level, local linkages, which usually correspond to polymorphisms in *cis*-regulatory elements, tend to be more stable across conditions, such that they are more likely to show the same effect or the same direction of effect across conditions. Distant linkages, which usually correspond to polymorphisms influencing *trans*-acting factors, are more condition-dependent, and often show effects in different directions in the two conditions. We characterized a locus that influences expression of many growth-related transcripts, and showed that the majority of the variation is explained by polymorphism in the gene *IRA2*. The RM allele of *IRA2* appears to inhibit Ras/PKA signaling more strongly than the BY allele, and has undergone a change in selective pressure. Our results provide a broad overview of the genetic architecture of gene–environment interactions, as well as a detailed molecular example, and lead to key insights into how the effects of different classes of regulatory variants are modulated by the environment. These observations will guide the design of studies aimed at understanding the genetic basis of complex traits.

## Author Summary

Individuals frequently encounter different environmental conditions, and the physiological and behavioral responses to these conditions can depend on an individual's genetic makeup. This phenomenon is known as gene–environment interaction. For example, individuals who are infected with the Plasmodium falciparum parasite are susceptible to malaria, but not if they carry the sickle-cell allele of hemoglobin. The general properties of gene–environment interaction are poorly understood, and a better understanding is essential if individuals are to make informed health choices guided by their genomic information. We have investigated gene–environment interaction on a genomic level, characterizing its role in over 4,000 traits at once by investigating natural variation in yeast gene expression. We compared lab and vineyard strains of yeast growing in two conditions (glucose and ethanol as carbon sources) in which they adopt two different metabolic states: fermentation and aerobic respiration, respectively. We show that gene–environment interaction is a common phenomenon, describe how different classes of genetic variants affect the nature of the interactions, and provide detailed molecular examples of interactions.

## Abstract 3

We show that gene-environment interaction is a common phenomenon in the regulation of gene expression, we describe how different classes of genetic variants affect the nature of the interactions, and we provide detailed molecular examples of interactions.

## Introduction

We are rapidly approaching an age when genomic sequence will be used to inform life decisions. The extent to which lifestyle choices will change how our genetic blueprint is expressed, however, depends on the presence of gene–environment interactions: the phenomenon where the effect of a genetic variant differs in multiple environments. In humans, gene–environment interactions have been reported for many diseases, including heart disease (reviewed in [1]), depression [2], and cancer [3]. More often, however, studies either fail to see these effects or they are difficult to reproduce. Studies in model and agricultural organisms have been more promising, particularly in experimental crosses where gene–environment interaction can be studied more easily on a genome-wide level, as opposed to only targeting candidate genes. When quantitative trait linkage analysis has been performed for the same trait in multiple environments, different loci are often found in the different environments [4], and when tested explicitly, these loci often display gene–environment interaction [5,6]. However, these studies are limited in their scope because they either examine one or a few phenotypes or restrict their analysis to loci that were initially found within a single condition. In humans, as well as in experimental systems, tracking down the molecular mechanisms of gene–environment interaction has proved difficult.

Recently, gene transcript abundance has been used as a model to study the genetics of thousands of quantitative traits at a time [7,8]. The studies have targeted organisms such as yeast [9], humans [10–13], mice [14–16], rat [17], and *Arabidopsis* [18]. Because so many traits can be studied at once, these studies have been able to elucidate trends in difficult-to-study phenomena, such as gene–gene interaction [19,20] and genetic complexity [21].

Gene–environment interaction recently has been studied using transcript levels in yeast [22] and in worms [6]. Landry et al. (2006) compared six strains in three conditions and found 221 transcripts showing gene–environment interaction [22], suggesting that there is much phenotypic diversity at the strain level in how yeast responds to its environment. Li at al (2006) mapped quantitative trait loci responsible for expression differences in two different temperatures in Caenorhabditis elegans [6]. They identified 197 linkages that showed gene–environment interaction, suggesting that studying gene–environment interaction of expression phenotypes using model organisms in controlled conditions should prove fruitful.

We have used a large family of yeast segregants to characterize gene–environment interaction on a global scale. Two parental strains (BY and RM, see Materials and Methods) and 109 segregant strains from a cross between the parental strains were grown in two conditions—glucose and ethanol as carbon sources—and expression profiled using microarrays. When growing in glucose, yeast predominantly ferment glucose to ethanol. When the cells run low on glucose, they switch to a primarily respiratory state in which they metabolize ethanol [23]. The transcriptional state of the cells changes dramatically during this period [24,25], with transcripts influencing growth and respiration particularly affected. This difference in the metabolic state may change how genetic variants influence traits, resulting in the observation of gene–environment interaction. By studying thousands of such traits, we sought to characterize the general genetic architecture of gene–environment interaction, and by using the molecular tools available in yeast, we were able to characterize some of the variants involved at the gene level.

## Results

Gene–environment interaction occurs when the phenotypic effects of genotype and environmental condition are not independent. For instance, a transcript could show a large difference in expression between two strains in glucose, but show no difference between these strains in ethanol. Thus, the effect of the genotype depends on which condition is tested. We characterized gene–environment interaction by two complementary approaches. First, we investigated gene–environment interaction on a strain level by comparing the two parental strains. Second, we used 109 segregants from a cross between the parents to characterize gene–environment interaction on a locus level.

### Strain–Condition Interaction Is Common in Parental Strains

**Figure 1:**
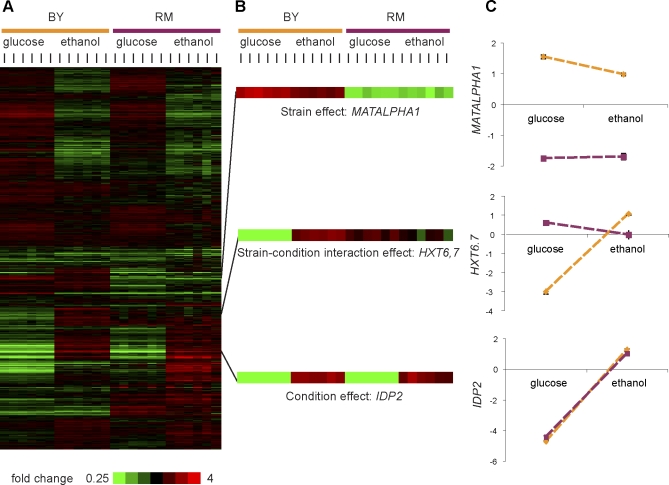
Strain, Condition, and Strain–Condition Interaction Effects in Parental Strains Six replicates of each parental strain (BY and RM) were expression profiled in each condition (glucose and ethanol), and 4,342 transcripts with high-quality data were tested for strain, condition, and strain–condition interaction effects using two-way ANOVA. (A) Clustergram of all 24 arrays and 4,342 transcripts. Note the high reproducibility of the sets of biological replicates. (B) Three transcripts are highlighted as strong examples of effects of strain (*MATALPHA1*), strain–condition interaction (*HXT6,7*), and condition (*IDP2*). (C) Two-factor plots with strain indicated in color (BY = orange, and RM = purple). The average of six values is indicated by each point, with error bars indicating the standard error. When no error bars are visible, the standard error was smaller than the point used to plot the average.

**Figure 2:**
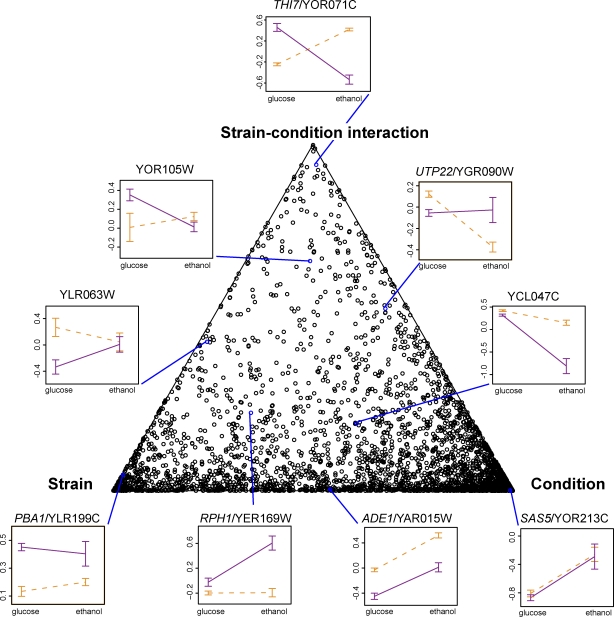
Distribution of Proportion of Variance Explained in Parental Strains The relative proportion of strain–condition interaction, strain, and condition variance is shown for all transcripts in the parental strains in which these variances add up to at least 50% of the total variance (3,219 of 4,342 transcripts; 74%). If a point is at a vertex of the triangle, the relative proportion of variance due to the labeled factor is 1, and the proportion decreases to zero along the line from the vertex to the midpoint of the opposite side. Insets show two-factor plots (reaction norms) for representative transcripts. The average for the BY strain is in orange, the average for the RM strain is in purple, and error bars indicate standard deviations. Transcript levels are log2 ratios versus the common reference.

We characterized the extent of gene–environment interaction influencing transcriptional phenotypes in the parental strains BY and RM by growing six independent cultures of each strain in glucose and in ethanol, and measuring gene expression with microarrays (Figure 1 and Dataset S1). We determined the influence of strain, condition, and the interaction between strain and condition (here we use strain–condition interaction to distinguish effects due to overall strain differences in genetic background, as opposed to a specific gene or locus) by using analysis of variance (ANOVA). Of the 4,342 transcripts with high-quality data, 2,037 (47%), 2,996 (69%), and 3,448 (79%) showed significant effects due to strain–condition interaction, strain, and condition, respectively (see Figure 1B and 1C for strong examples and Table S3 for a full list). Transcripts were often influenced by multiple factors (Figure S2A). We detected ten times as many transcripts showing strain–condition interaction as has been previously reported for gene expression in yeast [22]. This result is primarily due to the number of replicates we performed (six); an analysis with two replicates detected a number of interactions similar to the previous report. Strain–condition interaction effects accounted for 9% of the total variance explained over all transcripts, whereas strain and condition effects were larger, explaining 21% and 36% of the variance, respectively. The importance of each of these factors varied for individual transcripts, with each factor playing a dominant role for a subset of transcripts (Figure 2). Genetic correlations of transcript levels between the two conditions were on average low (mean genetic correlation = 0.26), with transcripts that show significant strain–condition interaction having lower genetic correlations than those that do not (−0.07 vs. 0.56, respectively; Mann-Whitney *p* < 10^−15^). These genetic correlations are lower than those observed for outbred livestock populations [26,27]. Thus, as previously reported, strain [9] and condition [25] effects profoundly influence gene expression, but interaction effects between the two are also important.

### Linkage Analysis Yields Loci Responsible for Gene–Environment Interaction

**Figure 3:**
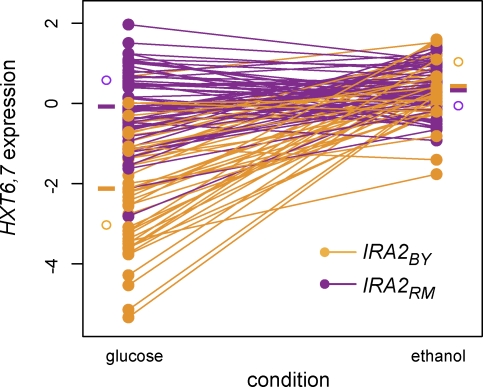
Interaction between Condition and *IRA2* Genotype in Expression of *HXT6,7* *HXT6,7* shows gene–environment interaction with the region containing *IRA2*. Phenotypes for 109 segregants are split by whether they inherited the BY allele (orange) or RM allele (purple) of the *IRA2* region. For each segregant, the expression values in glucose and ethanol are plotted and connected by a line. The difference between the expression values in ethanol and glucose (or the slope across conditions) is the phenotype that is used to find loci that show gene–environment interaction. In this case, segregants that inherit the BY allele show low expression *of HXT6,7* in glucose, but higher expression in ethanol. Segregants that inherit the RM allele, however, express *HXT6,7* at the higher level in both glucose and ethanol. Horizontal bars to the left and right indicate the mean phenotypic values for all segregants carrying a particular allele in a condition. Open circles indicate the mean phenotypic value for the parental strains in each condition.

To understand the genetic basis of gene–environment interaction, we measured transcript expression in 109 segregants derived from a cross between BY and RM [21]. Each of the 109 previously genotyped segregants was expression profiled in both glucose and ethanol conditions on microarrays (Dataset S2). We performed linkage analysis on the transcript levels within each condition, and 3,997 and 3,489 linkages were observed in glucose and ethanol, respectively (Figure S1 and Table S4). We determined loci that show gene–environment interaction (*gxe*QTL) by performing linkage analysis on the difference between conditions (for each segregant, the difference between conditions is the difference between expression in ethanol and expression in glucose; this type of measurement is also known as plasticity [28] and is similar to environmental sensitivity [29] (see Text S1)). The difference between conditions was calculated for each segregant for 4,482 transcripts with high-quality data (see Materials and Methods), and subjected to linkage analysis with 2,894 markers spaced throughout the genome. There were 1,382 transcripts that showed gene–environment interaction with at least one locus (total of 1,555 linkages) at a genome-wide 5% false discovery rate (FDR), as determined by permutation (see Figure 3 for an example). Transcripts that showed strain–condition interaction in the parents were more likely to show at least one linkage in the segregants (relative risk [RR] = 1.8, 95% confidence interval [CI] = 1.6–1.9, χ^2^ = 154, *p* = 3 × 10^−35^), but there was not a one-to-one correspondence. For some transcripts, effects were revealed in the segregants that were not observed in the parents. For others, we were unable to map specific loci responsible for differences observed in the parents. Some of this may due to differences in power, but because of genetic complexity, we do not expect to map a locus for all transcripts that show significant genetic or gene–environment interaction effects in the parent strains. Previous work has also shown that effects that are revealed only in the segregants (transgressive segregation) are common in the genetic regulation of gene expression within a condition [21]. We think that these phenomena are likely to play a role here as well.

### Local versus Distant Linkages

Since transcripts derive from open reading frames (ORFs) with physical locations in the genome, we can make a distinction between linkages that are physically near the ORF (local) and those that are far away from the ORF (distant). Local linkages are often due to variation at *cis*-acting sites [30–32], whereas distant linkages likely result from variants that influence a *trans* intermediate, such as those resulting in a change in protein function or concentration. Most transcripts linked to distant loci. However, for *gxe*QTL, this was especially pronounced. *gxe*QTL were less likely to be local, with 172/1,555 (11%) of all *gxe*QTL being local, as compared to linkages within either condition, where 22%–25% of all linkages were local (Table 1). This result agrees with previous reports that either directly addressed interaction or observed differences between conditions [6,10,15–17,33,34], and suggests that changes in *cis*-regulatory sites are less condition dependent than those influencing *trans*-acting factors.

**Table 1:**
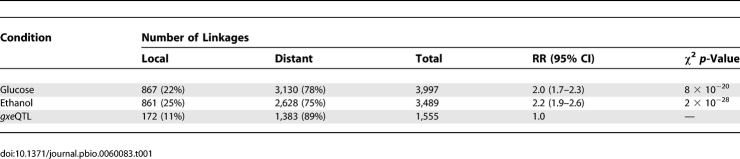
*gxe*QTL Are Less Likely to Be Local

### Distant Peaks

**Figure 4:**
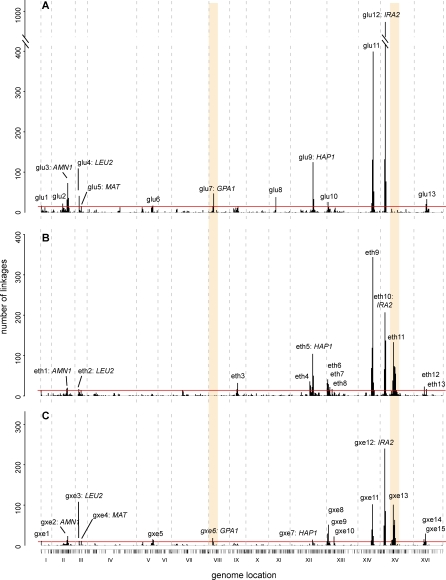
Expression Linkage Peaks Can Change between Conditions For linkages in glucose (A), in ethanol (B), and *gxe*QTL (C), the number of distant linkages falling in 10-cM large genomic bins is plotted. On the *x*-axis is the genomic location of each bin, with tick marks indicating the location of individual markers and roman numerals indicating chromosome number. We identified bins with a total number of linkages that is unlikely to occur by chance (this threshold is indicated by a red line; see Materials and Methods for details). Such bins located directly adjacent to each other were collapsed to a single peak. Peaks are labeled with a number when the underlying gene is unknown and additionally by a gene name when a polymorphism in the gene has been shown to be associated with expression phenotypes that linked to the region in at least one condition. For several regions, differences between distant linkages in glucose and distant linkages in ethanol are reflected in the *gxe*QTL peaks. Two regions with striking effects are highlighted in peach. On chromosome 8, a peak overlapping with *GPA1* is present in glucose, but absent in ethanol, and this difference is detected as gene–environment interaction. On chromosome 15, a peak with no known regulator is absent in glucose, present in ethanol (eth11), and shows gene–environment interaction (gxe13).

As has been previously reported in mouse [14–16] and yeast [9], distant linkages cluster in a small number of regions. We divided the genome into 10-cM–sized bins and counted the number of distant linkages observed in glucose, ethanol, or *gxe*QTL that fell within each of the bins (Figure 4). The majority of distant linkages fell into bins with a significant excess of linkages, with 81% (1,126/1,383) of distant *gxe*QTL occurring in 31/563 bins, or 5.5% of the genome. Significant bins that were located immediately next to each other were merged into a single peak to form 13, 13, and 15 peaks for glucose, ethanol, and *gxe*QTL, respectively (Figure 4 and Table S1). Some of these regions coincided with loci that have been characterized at the gene level, including *AMN1* [35], *GPA1* [35], *MAT* [19], *HAP1* [9], and *IRA2* (this paper).

Peaks identified in glucose did not coincide with those in ethanol (Figure 4). Thirteen peaks were found in each condition, but only seven were found in both. Two large peaks are found for *gxe*QTL, but are only observed within one condition, suggesting that the polymorphism likely acts in one condition, but not the other. We highlight these as striking examples of differences in genetic regulation across conditions. The first of these colocalizes with the gene encoding Gpa1, the G-protein alpha subunit of the pheromone response signaling pathway [36,37], which contains a polymorphism previously associated with transcripts involved in pheromone response [35]. In glucose, 49 transcripts show distant linkage here, whereas in ethanol, there are no distant linkages, suggesting that the polymorphism in *GPA1* does not influence these transcripts in ethanol. When we asked where these transcripts linked in ethanol, we found that 12 showed linkage to eth12, a peak that is also enriched for genes involved in the pheromone response. Given the overall rates of linkage to both loci, we would expect less than one overlap by chance, and the observed overlap of 12 genes is highly significant (Fisher exact *p*-value = 8 × 10^−19^). Within this region lies *DIG1*, which codes for an inhibitor of the pheromone response/invasive growth transcription factor Ste12 [38,39]. Allele replacements of *DIG1* in both backgrounds show that variants in *DIG1* make a major contribution to the effects at this locus (Figure S3). This example highlights how environmental differences can change the relative importance of genetic variants in the same pathway, resulting in different genes being more important in one or another condition.

Another example of a condition-specific distant peak is provided by the eth11/gxe13 peak on chromosome 15 (160–220 cM). A total of 386 transcripts show distant linkage to this locus in ethanol, whereas in glucose, only ten show distant linkage. In contrast to most other peaks, the transcripts linking here do not show functional enrichment for gene ontology (GO) terms. Targets of similar signaling pathways should share transcription factor binding sites, even if the functional implications of the pathway are unknown. To determine whether we could find enrichment for specific transcription factor binding sites, we split this group of transcripts into four groups, depending on whether they were up or down-regulated by condition and genotype. We used transcription factor binding site data derived from experimental binding as well as species conservation [40] to determine whether any of these gene lists were enriched for experimentally confirmed as well as potential transcription factor binding sites. Those genes that were expressed higher in glucose and in the segregants carrying the RM allele were enriched for sites where *MSN2* and *MSN4* target sequence was conserved (*MSN2 p* = 2 × 10^−7^, *MSN4 p* = 3 × 10^−6^), but not for sites that have been shown to experimentally bind *MSN2* or *MSN4*. Experimental binding of *MSN2* and *MSN4* was tested in a variety of conditions [41], but these did not include ethanol, and so these targets may reflect an additional functional role for these transcription factors. Those transcripts that show lower expression in ethanol and higher expression in segregants with the BY allele are enriched for *CIN5* binding sites that are both conserved and show experimental binding in the conditions tested (high and moderate hyperoxia, enrichment *p* = 6 × 10^−5^). *CIN5* is located in this region, shows local linkage with gene–environment interaction, and contains 17 nucleotide changes in the 500 bp upstream of start (two to three are expected on average, given an upstream polymorphism rate of 0.005 [30]), in addition to five coding single nucleotide polymorphisms (SNPs) (two nonsynonymous), making it an excellent candidate for regulating a subset of these transcripts. *CIN5* may not be the regulator for all transcripts linking here—because the peak is very wide, it may contain multiple variants.

### Condition Specificity of *gxe*QTL

After examining large differences in distant peaks, we sought to more generally and rigorously describe how the effect of a locus changes across conditions. Comparisons of *gxe*QTL with linkage results for transcript levels in glucose or ethanol showed that only 965/1,555 (62%) *gxe*QTL also reached genome-wide significance levels in at least one condition (see Figure S2B for full Venn diagram). However, for a test of whether a specific marker that represents a *gxe*QTL is also linked to the expression level of the corresponding transcript in one of the conditions, setting a genome-wide significance threshold is too conservative. We tested for linkage between the most significant marker at each of the 1,555 *gxe*QTL and expression of the respective transcripts in glucose and in ethanol, lowering the threshold to a FDR of 5% for testing a single marker for each trait, as obtained through inspection of the *p*-value distribution with QVALUE [42]. All 1,555 *gxe*QTL showed an effect in at least one condition, with 265/1,555 (17%) showing an effect only in glucose, 327/1,555 (21%) showing an effect only in ethanol, and 963/1,555 (62%) showing an effect in both conditions. The fact that all *gxe*QTL showed linkage to the expression phenotype in at least one condition is not surprising, as a difference in how the genotypes differ between two conditions mathematically requires a change in the mean phenotypic value in at least one condition. The QVALUE method also provides an estimate of the proportion of loci found within a condition that show gene–environment interaction. We inspected the distribution of all *p*-values for linkage between the most significant marker in one condition and the difference in expression between the conditions, and found that 77% and 76% of loci in glucose and ethanol, respectively, also influence the difference between conditions.

**Figure 5:**
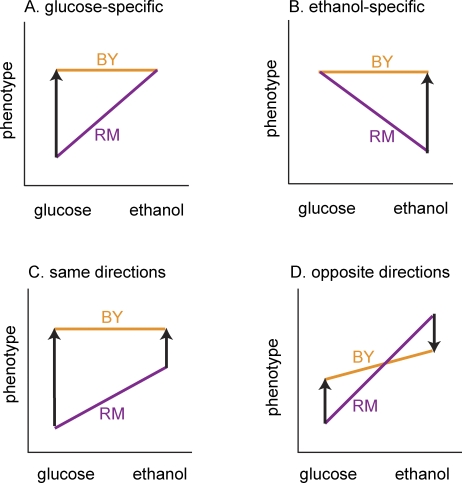
A Schematic of Different Types of Gene–Environment Interaction Effects Gene–environment interaction can occur in a number of different ways. In these plots, different alleles at a locus (these could also be different strains) are indicated by solid lines colored either orange or purple. These solid lines connect the two measurements that were taken in the two conditions for ease of visualizing the difference between conditions. When different alleles show a significant difference in one of the conditions, a black arrow connects the two measurements and indicates the difference between the orange and the purple alleles. In (A) and (B), the locus shows condition specificity and only has an effect in one of the conditions. In (C) and (D), the locus shows a significant effect in both conditions. In (C), the difference between the alleles is in the same direction in the two conditions. In (D), the difference between the alleles is in the opposite direction in the two conditions.

Since promoters often define when and where transcripts get expressed, it might be thought that when they do show gene–environment interaction, variants influencing *cis*-acting regions would be more likely to be condition specific. We did not observe this relationship between local and distant linkages, however. Distant linkages were significantly more likely to be condition specific than local linkages, as 546/1,383 (40%) of distant linkages were significant in only one condition, as compared to 46/172 (27%) of local linkages (Table 2 and see Figure 5 for a schematic). Thus, local linkages are overall less dependent on the environment, and when they do show gene–environment interaction, their effects are less likely to be restricted to a specific condition.

**Table 2:**
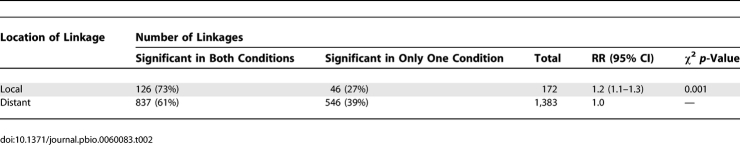
Local *gxe*QTL Are More Likely to Have Significant Effects in Both Conditions

**Figure 6:**
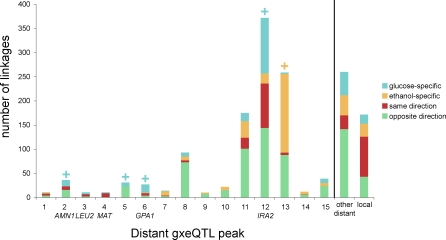
*gxe*QTL Peaks Show Variation in Condition-Specific Behavior For each *gxe*QTL peak, the number of linkages that are glucose specific (blue), ethanol specific (orange), active in both conditions, with the same direction of effect (red), and active in both conditions, with opposite direction of effect (green). On the right for comparison, all distant linkages that did not fall into a peak are combined into one group, and all local linkages are combined into one group. Significant results from the test for enrichment of glucose specific (blue plus [+] signs) or enrichment of ethanol specific (orange plus [+] sign) are indicated above the bars.

We investigated whether individual hot spots showed condition specificity by comparing the proportion of *gxe*QTL in each hotspot that were glucose specific or ethanol specific to the overall rates in all distant *gxe*QTL using a hypergeometric test (Figure 6). Five hot spot regions showed altered proportions of condition-specific *gxe*QTL: peaks gxe2 (*AMN1*), gxe5, gxe6 (*GPA1*), and gxe12 (*IRA2*) were enriched for glucose-specific linkages, whereas peak gxe13 was enriched for ethanol-specific linkages. These results agree with the observation that distant peaks change between conditions, but provide a more quantitative result that indicates that some distant loci show differential effects between conditions. This may be due to changes in the activity states of the proteins and pathways involved as conditions change, or it could also be due to epistasis that masks the effects of the polymorphisms in the alternate condition.

### Direction of Locus Effect

Loci showing gene–environment interaction that have effects in both conditions may act by increasing expression in one condition and decreasing expression in the other or by affecting expression in the same direction in both conditions, but to a different extent (see Figure 5 for a schematic). Of those *gxe*QTL that had an effect in both conditions (963/1,555), the effects were in opposite directions 72% of the time. This phenomenon was dependent on the type of locus, as local *gxe*QTL were more likely to act in the same direction in both conditions (66%), whereas distant gxeQL were more likely to act in opposite directions (78%) (Table 3). The majority of the loci that had effects in opposite directions did not reach genome-wide significance in either condition (463/697). Exclusion of loci that were not genome-wide significant in at least one condition only slightly reduced the association between the type of locus and the direction of effect (RR = 2.3 vs. 3.0). The high rate of distant loci acting in opposite directions is not due to an individual peak, as most distant peaks, as well as the set of all linkages that fall outside of peaks, have a high proportion of linkages that act in opposite directions (Figure 6). This pattern is consistent with the result that local *gxe*QTL are less likely to show gene–environment interaction overall and less likely to be condition specific.

**Table 3:**
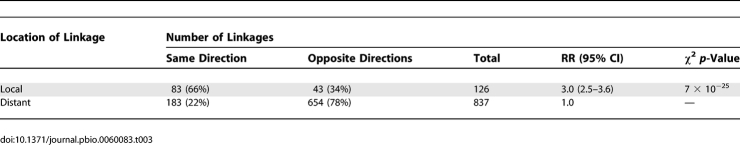
Among *gxe*QTL, Local Linkages Are More Likely to Have Effects in the Same Direction in Both Conditions

### Polymorphism in *IRA2* Is Responsible for gxe12

**Figure 7:**
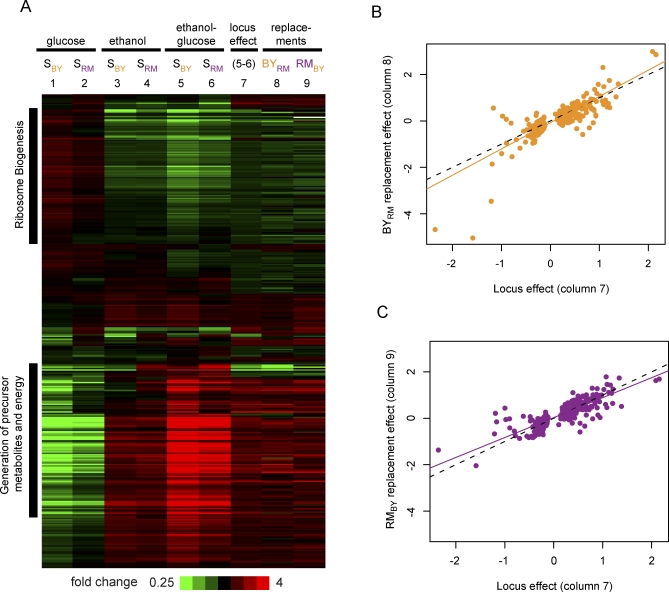
Polymorphism in *IRA2* Contributes to Environmentally Dependent Phenotypic Variation (A) Expression values for 370 transcripts with *gxe*QTL confidence intervals that overlap *IRA2* are shown. Average expression values for segregants (S) carrying either allele in glucose, in ethanol, and for the difference between ethanol and glucose are shown in columns 1–6. The overall effect of the locus between conditions (locus effect) is shown in column 7, which is the difference between columns 5 and 6. The effects of the replacements are shown in column 8 and 9. The difference between conditions in each replacement was compared to the difference observed in the appropriate parental background strain (BY in column 8, and RM in column 9). The similarity of columns 8 and 9 to column 7 indicates that polymorphism in the replaced region recapitulates the effect of the locus well and shows that polymorphism in *IRA2* is functionally responsible for determining how these transcripts differ between conditions among the segregants. (B) Scatterplot of the locus effect (column 7 in [A]), versus the effect of introducing *IRA2*-RM into the BY background (column 8 in [A]). Each point represents a transcript; orange line is the best fit by linear regression. The black dotted line indicates *y* = *x*. The slope of the regression line is 1.1 (95% CI 1.05–1.21) with a correlation of 0.84 (70% variance explained), and a permutation *p*-value of 0.002. (C) Scatterplot of the locus effect (column 7 in [A]), as compared to the effect of introducing *IRA2*-BY into the RM background (column 9 in [A]). Symbols and lines as in (B). The slope of the regression line is 0.86 (95% CI 0.80–0.92) with a correlation of 0.83 (69% variance explained), and a permutation *p*-value less than 0.001.

We further investigated peak gxe12, located at chromosome 15 50–80 cM, which we observed to be enriched for transcripts that show glucose-specific effects. A total of 372 transcripts link here distantly, and many are generally involved in energy metabolism and growth (Figure 7A). Most of these transcripts show large changes between glucose and ethanol, but the allele that the segregant inherits determines the strength of the change. The transcripts that are repressed in ethanol are enriched for ribosomal biogenesis and assembly (*p* = 8 × 10^−13^), whereas those that are activated in ethanol are enriched for generation of precursor metabolites and energy (*p* = 1 × 10^−6^). *IRA2* is a strong candidate for the regulation of these transcripts. Ira2 is an inhibitor (GTPase activating protein) of the Ras proteins, which mediate the cellular response to glucose via the Ras/PKA pathway [43]. Components of this pathway are highly conserved across species, and Ira2 is a homolog of the neurofibromin tumor suppressor in humans [44]. The *IRA2* coding region is highly polymorphic, with 87 SNPs and a single 3-bp indel differing between the strains, resulting in 61 synonymous changes, 26 nonsynonymous changes, and one RM insertion. Of the 26 nonsynonymous changes, BY carries the ancestral amino acid (Saccharomyces paradoxus) in 20 cases, and RM carries the ancestral amino acid in four cases, with the remaining two different from S. paradoxus in both strains.

To determine whether polymorphism within *IRA2* is responsible for the observed linkages, we generated allele replacement strains that carried the coding region of each parent strain in the background of the other parent. We felt that targeting the coding region was appropriate because the expression of *IRA2* has not shown local linkage [21]. We expression profiled the replacement strains in glucose and ethanol, and compared the effect of the replacement to the effect of the locus observed in the segregants (Figure 7B and 7C). We observed high correlations between replacements in both parental backgrounds and segregant effects for transcripts showing gene–environment interaction, and the regression slope for both was close to 1. Many transcripts link to this region in glucose and ethanol (1,159 and 410, respectively), and *IRA2* polymorphisms also contribute to the variation in transcript levels within these conditions (Figure S4). Thus, polymorphism in *IRA2* appears to be the major contributor to the difference in expression between conditions as well as to transcript abundance in each condition for many transcripts linking to this region. This is consistent with coding variants in Ira2 influencing transcript levels via changes in the signaling state of the Ras/PKA pathway.

**Figure 8:**
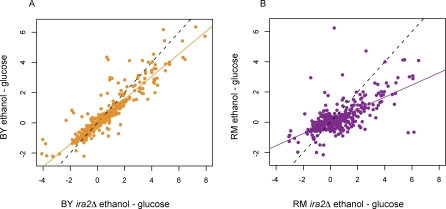
The BY Allele of *IRA2* Is More Similar to a Knockout Than the RM Allele of *IRA2* In order to assess the role of different alleles of *IRA2*, this gene was knocked out in both parental strain backgrounds. For all transcripts with *gxe*QTL in the region containing *IRA2*, we plotted the change in expression across conditions (ethanol–glucose). (A) Scatterplot of the difference for the knockout (*x*-axis) versus the parental strain (*y*-axis) in the BY background. (B) Scatterplot in the RM background. The BY *ira2*Δ knockout is significantly different from the parental strain (regression slope = 0.75, 95% CI = 0.71–0.78), but the RM *ira2*Δ knockout shows an even larger difference from the parent strain (regression slope = 0.40, 95% CI 0.35–0.45). This indicates that in the conditions studied, the RM allele of *IRA2* is playing a larger role in determining how these transcripts differ between conditions.

To determine which allele had a stronger effect on the Ras/PKA pathway, we compared the *IRA2* alleles to their respective knockout strains. We knocked out *IRA2* in both parental strains, grew the resulting strains in glucose and ethanol, and then measured the transcriptional response, as compared to the parental strain (Figure 8). If the allele is active under the conditions that we tested, then the knockout should look different from the parental strain. We observed that both knockout strains were different from the parental strains, with linear regression slopes less than 1 (BY slope = 0.75, 95% CI: 0.71–0.78; RM slope = 0.40, 95% CI: 0.35–0.45), but the RM knockout showed a greater difference from the parental strain (Figure 8). Since knocking out *IRA2* in RM causes a bigger change than knocking it out in BY, this suggests that the RM allele of *IRA2* is better at inhibiting the Ras/PKA pathway in these conditions.

The BY allele of *IRA2* could be a poorer inhibitor because BY has lost some ability to inhibit or because RM has gained this ability. We took a sequence-based approach and compared *IRA2* coding sequences from the three sequenced S. cerevisiae strains (S288c, isogenic to BY; RM11-1a; and YJM789 [YJM]), with S. paradoxus as an outgroup. The S288c sequence is more similar to the ancestral S. paradoxus than either RM or YJM, indicating that RM *IRA2* is the more diverged allele. BY is more similar to the ancestral sequence, suggesting that RM has gained the ability to inhibit signaling more. However, since we do not know the specific causal variant(s), we are unable to rule out the possibility that a small number of polymorphisms that are not characteristic of the overall trend are responsible for a decrease in the BY allele's ability to inhibit.

If the RM allele has gained the ability to more strongly inhibit Ras/PKA signaling, then it might be expected to show signs of selection. We used the McDonald-Kreitman test to look for evidence of selection in *IRA2* by comparing rates of nonsynonymous and synonymous substitutions at polymorphic versus fixed sites within the three sequenced S. cerevisiae strains, as compared to the outgroup S. paradoxus. Of the sites that were fixed in S. cerevisiae, 133/882 (15%) were nonsynonymous, whereas within polymorphic sites, this rate was 2-fold higher (31/103, 30%). This difference is significant (Fisher exact *p* = 0.0001), suggesting that positive selection, or a relaxation of negative selection, has occurred in the S. cerevisiae strains. The RM strain appears to be contributing the most to the nonsynonymous rates, as the ratio of nonsynonymous to synonymous changes along the RM branch is the highest within the strains. Thus, the RM allele appears to have experienced a change in selection pressure in the past. Further analyses of a variety of yeast isolates should help elucidate the evolutionary history of this locus.

## Discussion

Here, we have used yeast gene expression as a model system to describe gene–environment interaction at strain, locus, and gene levels. We showed that 2,037 transcripts were jointly dependent on strain and condition in two parental strains. Then, we performed linkage analysis on the difference in transcript levels across conditions with 109 segregants, and identified 1,555 *gxe*QTL. The high number of *gxe*QTL that we detected has allowed us to make some general observations. We have shown that local and distant linkages differ dramatically in how they act across multiple conditions. Local linkages appear to be more stable: they are less likely to be dependent on the environment, and even when they are, they are more likely to have an effect in both conditions, with the direction of effect often being the same. Distant linkages, on the other hand, are more volatile: they are more likely to be dependent on condition and to show an effect in only one condition. Entire distant peaks can change across conditions, and when they do have an effect in multiple conditions, distant loci are more likely to act in different directions. Finally, we characterized the gene responsible for influencing the largest gene–environment interaction distant peak, *IRA2*. We showed that the RM allele of *IRA2* is a stronger inhibitor of Ras/PKA signaling than the BY allele in the conditions that we tested, and that this locus has experienced a change in selective pressure in RM.

Previous studies have reported that local linkages are more consistent than distant linkages across conditions and experiments, including worms in different temperatures [6], different tissues in mice [15,16,33], and in the reproducibility of transcript linkages in human studies [10,34], indicating that this pattern is likely to extend beyond yeast. Since local and distant linkages are likely to differ in how they influence traits on a molecular level, we can speculate as to how they show differences in condition dependence. Although we do not know most of the causative polymorphisms involved for each type of linkage, local linkages show increased rates of polymorphism in 5′ and 3′ noncoding regions and high rates of allele-specific expression [30]. Thus, we feel comfortable treating the two groups as distinct entities: local linkages are likely to be enriched for variants that directly influence transcript levels via changes in *cis*-regulatory sites, whereas distant linkages typically influence levels via a protein intermediate (*trans* factors). In a *cis*-regulatory site that interacts with a binding protein to directly increase or decrease transcript levels, mutations can either disrupt or enhance binding, but are unlikely to be able to do both in a condition-specific manner. If the binding protein is an activator, a loss of binding mutation will result in lower transcript levels in all conditions where the activator is present, and will show no change when the activator is absent, resulting in either a condition-specific pattern or in a pattern in which the locus has the same effect or the same direction of effect in both conditions (Figure S5A). Other than the case where a single polymorphism destroys a site for one binding factor while creating a site for another, it is less clear how one *cis*-regulatory mutation could be associated with effects in opposite directions, although one might imagine more complicated scenarios with either multiple linked *cis*-regulatory polymorphisms or transcription factors that are able to act as both activators and inhibitors. On the other hand, distant variants that influence protein intermediates have the potential to interact with many proteins, depending on the milieu present in the cell in a given condition (Figure S5B). A single variant may be able to activate transcription in one condition and repress it in another, resulting in a change in direction of effect.

The frequent occurrence of locus effects in opposite directions in the two conditions is surprising. One possible explanation is multiple linked polymorphisms. Distant loci are much larger targets for variation than *cis*-regulatory regions [45], but this could occur in both contexts. Multiple compensatory mutations could accumulate at loci and, depending on the condition, could compensate differentially. The mean phenotype would be stable over conditions, yet the direction of the effect within a condition could vary (Figure S5C). One example is suggested by the gxe11 peak on chromosome 14, where multiple polymorphisms that influence sporulation [46,47] and high-temperature growth [48] have been characterized, and some act in the opposite direction of the overall locus effect. At least one of these alleles (*MKT1* D30G) is at least partially responsible for the *gxe*QTL in this region (Figure S6). Further characterization of the polymorphisms involved at these loci should help elucidate the underlying mechanisms behind this phenomenon.

A practical implication of the observation that distant loci often act in opposite directions in the two conditions is that such loci may be inherently difficult to detect in experiments where condition is not controlled, as when different tissues of multicellular organisms are mixed or when nonexperimental organisms (including humans) that experience different unmeasured environments are studied. This is because when conditions are not controlled, effects of opposite direction will cancel each other out, resulting in no overall association between the trait and the locus. This also has implications for selection, as these loci may be hidden from selection in organisms that experience fluctuating environmental conditions. The detection of loci that act in opposite direction is additionally complicated by our observation that the majority of these loci did not reach genome-wide significance in either condition alone, emphasizing the importance of using methods to directly test for gene–environment interaction without prior reliance on linkage in a single environment.

One general implication of our results for studies in other species, including humans, is that many genetic effects on most traits are likely to be detected without testing for gene–environment interactions, provided that the relevant environmental factors are known and controlled either experimentally or statistically. However, analyses that ignore gene–environment interactions introduce strong biases with regard to the types of loci that are detected. Moreover, gene–environment interactions play a dominant role for a minority of traits. We have studied the prevalence and importance of gene–environment interactions in a single-cell organism grown in two very different and precisely controlled environmental conditions. Our focus on transcript levels as quantitative traits allowed us to study a very large number of traits simultaneously and to delineate general patterns, as well as to provide detailed molecular examples of loci that show gene–environment interactions. The quantitative details would undoubtedly differ if different species, environments, and phenotypes were studied. It is possible that many environmental differences experienced by humans may be less drastic than those between growth on glucose and ethanol are for yeast. However, some environmental differences (for example, exposure to pathogens or shift from traditional to modern diets) can have a dramatic effect on health. Our detailed studies in a model organism provide examples of the types of effects that may be expected in humans, and thereby inform practical study design. Understanding the subset of human genetic variants whose phenotypic effects are modifiable by the environment will be key in making full use of personal genomic variation.

## Materials and Methods

### Culture conditions.

The segregants used in this study have previously been expression profiled in condition similar to the glucose condition used here [21]. However, in order to make conditions as similar as possible for this study, with only the carbon source differing, these experiments were repeated alongside the experiments in ethanol. Yeast strains were grown in media consisting of 6.7g/l Yeast Nitrogen Base (Sigma Y0626), 100 mg/l leucine, 100 mg/l lysine, 20 mg/l uracil, and one of the following: 1% (v/v) ethanol or 2% (w/v) glucose. All growth was performed at 30 °C. After streaking to rich medium (YPD) plates from the freezer, cells were pregrown in 5 ml of the phenotyping medium overnight, while spinning on a rotor drum. Approximately 5 × 10^5^ (glucose) or 10^7^ (ethanol) cells were transferred to 15 ml of fresh medium in a 125-ml Erlenmeyer flask and grown overnight on a rotary shaker at 200 rpm. Cells were harvested at an optical density at 600 nm (OD_600_) between 0.36 and 0.40. Cells were collected (5 ml) at 30 °C by vacuum filtration, and the filters were immediately frozen in liquid nitrogen. Replicates of parental strains and parental strains used to generate the reference sample were grown interspersed with segregant strains such that the parental variation would reflect experimental variation throughout the experiment.

### RNA extraction and labeling.

RNA was extracted from the filters using a standard hot acid phenol method, followed by RNeasy cleanup (Qiagen). Samples were quality controlled with RNA 6000 Nano kit of the Bioanalyzer 2100 (Agilent). Samples were labeled with either Cy3 or Cy5 dye, using the Low RNA Input Linear Amp Kit (Agilent) with the modification that half reactions were used with a quarter of the recommended dye.

### RNA reference.

All samples were hybridized against the same common reference consisting of equal amounts of RNA from both parents (BY and RM) grown in both conditions (glucose and ethanol). Six independent replicates of each strain–condition combination were grown and harvested, for a total of 24 samples. RNA was isolated for each sample individually and then pooled, with an equal amount of RNA contributed by each sample. Multiple labeling reactions (24 for each dye) were pooled, and the same samples were used for all segregant and parental arrays. Experiments for knockout and allele replacement arrays used the same reference RNA sample, but a new labeling reaction.

### Hybridization and preprocessing.

Samples were hybridized to Agilent 11k yeast arrays, which are two-color, 60-mer oligo arrays with two arrays per slide, each containing spots for 6,256 transcripts, with some duplicated spots. In order to minimize experimentally induced bias, we randomized the order in which samples were processed, hybridizing glucose and ethanol samples at the same time and on the same slides. After hybridizing and washing per Agilent instructions, the arrays were scanned using an Agilent scanner and analyzed with Agilent's Feature Extraction software (versions 8.0–9.5). The arrays were uploaded into PUMAdb for processing (http://puma.princeton.edu/). Spots were considered good data if intensity was well above background and the feature was not a nonuniformity outlier. Additionally, transcripts were retained if data were present in all parental strains in the parental analysis or in at least 80% of the segregants in both conditions for the segregant analysis. We ultimately used 4,342 transcripts for the parental analysis and 4,482 transcripts for the segregant analysis.

### Parental analysis of variance analysis.

Six replicates of each parent (BY and RM) in each condition (glucose and ethanol) were grown, expression profiled, and labeled (half with Cy3 and half with Cy5). There were 4,342 transcripts with high-quality data: there were no polymorphisms within the probe sequence, and good data was present for each of the replicates. For each transcript, an ANOVA of the form

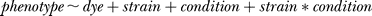
was performed in R using the aov function. In order to assess significance, the dataset was permuted with respect to strain and condition 1,000 times, retaining six values of each strain–condition combination, and repeating the ANOVA for each transcript. A FDR was calculated for a range of *p*-values for strain, condition, and strain–condition interaction effects separately. *p*-Value cutoffs of 0.04, 0.04, and 0.03 corresponded to FDRs of 5% for strain, condition, and strain–condition interaction effects, respectively.

### Genetic correlation.

Genetic correlation was calculated from the variance results of the ANOVA study using the following equation [49].

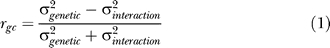



### Segregant linkage analysis.

Segregants were grown and expression profiled in both conditions, resulting in a total of 218 segregant arrays: 109 segregants each in glucose and ethanol. Samples were randomized with respect to dye, and all arrays were linearly adjusted for dye. Three phenotypes were used in linkage analysis: expression in glucose, expression in ethanol, and the difference in expression between glucose and ethanol (ethanol minus glucose for each segregant). Mapping a significant locus for the change across conditions is interpreted as gene–environment interaction. It can also be conceptualized as a paired test where the segregant's ethanol phenotype is effectively corrected for its phenotype in glucose.

Genotypes have been previously characterized [21], but we further refined the map by removing a set of 62 markers, corresponding to 13 Affymetrix gene regions, that were artificially inflating the map. When each gene region was removed, the total map distance (Haldane) decreased at least 25 cM. All but one of these gene regions corresponded to sequence that was duplicated in the genome, which would increase genotyping error. Our final set of markers consisted of 2,894 markers with an average spacing of 4 kb or 1.9 cM. Linkage analysis was performed using the nonparametric method of the qtl package in R [50], which is an adaptation from the Kruskal-Wallace test and is similar to that described by Kruglyak and Lander [51]. In addition to the genotypes of the markers, genotype probabilities were estimated at every centimorgan along the genome using the calc.genoprob function. Significance was assessed by permutation. For each permutation, segregants were assigned to a new array randomly, and linkage analysis on all transcripts was repeated. The permutation was performed ten times and the average number of transcripts showing linkage at a specific logarithm of the odds (to the base 10) (LOD) score was used to calculate a FDR. LOD scores of 3.25, 3.3, and 3.7 corresponded to FDRs of 4.9%, 4.9%, and 4.8% for glucose, ethanol, and the difference between condition phenotypes, respectively. We also calculated confidence intervals as LOD support regions that extend outward from the peak marker until the LOD score decreases by 1.5 LOD units [52].

### Local and distant linkage classification.

A linkage was classified as a local linkage if the confidence interval of the linkage overlapped with the region containing the open reading frame of the gene of interest (ORF region). The two markers flanking the region from 500 bp upstream of the start of the ORF to 500 bp downstream of the stop of the ORF, as defined by the *Saccharomyces* Genome Database, defined the ORF region. A distant linkage is any linkage that fell outside of the ORF region.

### Peaks of distant linkages.

The genome was broken into 10-cM bins, and the peak of each linkage in each condition (glucose, ethanol, or gene–environment interaction) was assigned to a bin. A bin was considered to have an excess of linkages if the number exceeded the number expected by chance by Poisson distribution, given the total number of distant linkages and Bonferroni correction for 563 tests (*p* < 9 × 10^−5^). These cutoffs were greater than 15 for glucose, 14 for ethanol, and nine for interaction. Significant bins that were located immediately next to each other were merged into a single peak. Peaks in different conditions were considered overlapping if any bin contained in one peak was contained in the other.

### Condition-specificity tests with QVALUE.

For each *gxe*QTL, we took the marker closest to the linkage peak and computed the LOD score for linkage of this marker to the transcript levels in glucose and in ethanol. We then converted the LOD score to a nominal *p*-value by comparison to permuted data. We created 1,000 random phenotypes and performed nonparametric linkage analysis on each. We translated LOD scores to *p*-values for a given marker by counting the proportion of the randomized phenotypes that had a LOD score as high or higher than that observed. By inspecting the *p*-value distribution of *gxe*QTL marker linkages in each condition using QVALUE software [42], we determined cutoffs that allowed us to call individual linkages significant at a FDR of 5% (*p* = 0.228 in ethanol and *p* = 0.22 in glucose). To estimate the proportion of linkages within a condition that also showed gene–environment interaction, we calculated *p*-values for the linkage between peak markers found within a condition and the difference in expression between conditions. The proportion of significant tests is 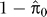 .

### Tests of association.

RR estimates and 95% CIs were calculated in R using the twoby2 command of the Epi package. Values of χ^2^ and *p*-values were calculated in Excel with expected values derived from row and column expectations. The Mann-Whitney test was performed in R using the wilcox.test command of the stats package.

### Probe polymorphism detection.

The RM genome was downloaded from the Broad Web site and aligned to the S288c genome (SGD). The sequence of each probe was found in the S288c sequence, and the corresponding sequence determined for RM. We were able to find 6,217 probes in the alignment, and of these, 5,029 have no polymorphisms at all, 747 have a single polymorphism or gap, and 438 have two or more strain differences. Missing probes were either in the mitochondrial genome or differed from the most recent reference sequence. The presence of even a single polymorphism was associated with an increased probability of apparent local linkage, as would be expected if there were differences in binding efficiencies of the alleles. We thus excluded them from all further analysis.

### Strain construction.

*IRA2* and *DIG1* replacement strains were generated by a two-step allele replacement method [53]. For example, *IRA2* was replaced with *URA3* in BY4724 [54] (*MATa ura3 lys2*) and RM11-1a [9] (*MATa leu2 ura3 ho::KAN*), generating *ira2Δ::URA3* knockout strains. New *IRA2* alleles were amplified by PCR with approximately 500-bp overlapping sequence and introduced into the appropriate background to replace *URA3*. See Table S2 for strain descriptions. Allele replacements were sequenced to ensure that no new mutations were introduced. For both replacements, the entire coding sequence was exchanged, but the extent that the 3′UTR polymorphisms were exchanged varied. Adam Deutschbauer kindly provided the *MKT1* D30G replacement strain in the BY4742 background [46].

### Sequencing.

The RM *IRA2* sequence was obtained from the whole-genome sequencing project at the Broad Institute (http://www.broad.mit.edu/annotation/genome/saccharomyces_cerevisiae/Home.html), with the exception of a small gap, which we sequenced using standard dideoxy methods. Replacement strains were also sequenced using standard dideoxy sequencing methods.

### Replacement analysis.

We quantified how well the *IRA2* replacement strains recapitulated the effect due to the locus in the segregants by comparing the locus effect to the replacement effect for all linking transcripts. For a given transcript, the locus effect is the difference between the BY change across condition (average of all segregants carrying the BY allele in ethanol minus the average of all segregants carrying the BY allele in glucose) and the RM change across condition (average of all segregants carrying the RM allele in ethanol minus the average of all segregants carrying the RM allele in glucose). For the replacement effect, the calculation is similar, except instead of segregant strains, we use the parental and replacement strains. Thus, for a single transcript, the replacement effect would be:

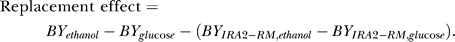



We then compared the locus effect to the replacement effect across all transcripts by calculating a Pearson correlation. We estimated significance of this correlation by randomizing the genotype at the marker nearest to *IRA2* and repeating the analysis 1,000 times. The *p*-value is the number of times that we got a correlation as high or higher than what was observed. A significant correlation indicates that gene that was replaced functionally influences the traits. We also analyzed this relationship by linear regression using the lm command in R. If the regression slope is close to 1, then it indicates that the gene is a major contributor to the phenotype.

### GO term enrichment.

Of the 4,482 transcripts that were analyzed in the segregants, 4,339 had GO terms associated with them. This set was used as a background for GO term enrichment. Hypergeometric tests were performed using GOLEM [55], and *p*-values were Bonferroni corrected for multiple testing.

### Transcript factor binding site enrichment.

Of the 4,482 transcripts that were analyzed in the segregants, 4,465 were used to look for enrichment of transcription factor (TF) binding sites. All nine “ORFs_by_factor” files that are provided by MacIsaac et al. on their Web site (available at: http://rd.plos.org/pbio.0060083) were used to look for enrichment of binding sites. These lists are constructed by creating lists of genes that contain a predicted TF site at three levels of conservation and three levels of experimental binding [40]. In order to be in a list, the site has to occur within the upstream intergenic region of the gene (K. MacIsaac, personal communication). Hypergeometric tests of enrichment were performed in GOLEM using modified input files in which each gene list corresponded to its own term. *p*-Values were Bonferroni corrected.

### McDonald-Kreitman test.

The McDonald-Kreitman test [56] was performed using the libsequence library's MK test [57] with sequences from RM, BY, YJM789 [58], and S. paradoxus [59]. Sequence trees and estimates of nonsynonymous to synonymous rates on individual branches were calculated in HYPHY [60].

## Supporting Information

Dataset S1 Parental Processed Data Log2 ratios (experimental/reference) values for all parental replicates. Data have been processed according to Materials and Methods, and only good data are reported.
(1.7 MB XLS)

Dataset S2 Segregant Processed Data Log2 ratios (experimental/reference) of segregant glucose and ethanol expression values. Data are processed according to Materials and Methods, and only good data are reported.
(6.6 MB XLS)

Figure S1 Linkage Map The linkage map was calculated with 109 segregants using the Haldane mapping function in R/qtl, with the chromosome and order of the markers determined by their physical position. Chromosomes are indicated by vertical lines, and markers are indicated by horizontal lines. The map covers a total of 5,545 cM.
(663 KB PDF)

Figure S2 Venn Diagrams of Parental and Linkage Analyses (A) The number of transcripts that show significant strain–condition interaction, strain, and condition effects in the parental strains.
(B) The number of linkages showing genome-wide level significance for gene–environment interaction, glucose, and ethanol. For each linkage, the marker closest to the peak of linkage was investigated in each condition. If the LOD score exceeded the genome-wide–level threshold, then the marker was considered significant in that condition. Since we are pooling results from different analyses, there are multiple linkages per chromosome. We randomly picked one of these linkages for the diagram and removed the rest. The totals within each condition do not add up to the total number of significant linkages reported because although a linkage can occur on the same chromosome in glucose and ethanol, they are not necessarily close enough that both markers are significant in both conditions, particularly when the scores are near the significance thresholds.
(116 KB PDF)

Figure S3 Polymorphism in *DIG1* Is Responsible for Expression of Linking Transcripts The relationship between the segregant effect and the replacement effect is shown for all transcripts that have a peak of linkage at the marker closest to *DIG1*. We chose to use the marker closest to *DIG1* to limit the proportion of transcripts that were being influenced by nearby peaks (gxe15). Each point represents a transcript. Solid lines indicate the best fit by linear regression (orange = BY, and purple = RM). The grey dotted line indicates *y* = *x*. Both lines are significantly different from 0, as indicated by the slopes.
(150 KB PDF)

Figure S4 Polymorphism in *IRA2* Is Responsible for Expression of Linking Transcripts within Conditions The relationship between the segregant effect and the replacement effect is shown for all transcripts that have confidence that overlap with *IRA2* are shown. Associations are shown for linkages in (A) glucose and (B) ethanol. Each point represents a transcript. Solid lines indicate the best fit by linear regression (orange = BY, and purple = RM). The grey dotted line indicates *y* = *x*. Linear regression slopes, confidence intervals, and *R* ^2^ values are shown for each line.
(283 KB PDF)

Figure S5 Potential Scenarios Leading to Gene–Environment Interaction (A) A scenario involving the loss of a transcription factor binding site across conditions that differ in the concentration (or activity) of the transcription factor. Depicted in the graph are two alleles, one with a mutation upstream of a start site (asterisk) and one without. Dotted lines indicate time points when measurements are taken. The activity at the locus is depicted below the graph for these two time points where the transcription factor (double circle), when present, is binding to the functional site and causing an increase in expression or no change. Expression levels are shown increasing from green to red, relative to the average expression.
(B) A scenario involving a transcription factor that is able to bind an activator or an inhibitor. An open circle indicates a protein capable of binding both factors, while the three-quarter circle indicates a protein unable to bind to either. The activator and the inhibitor vary in concentration depending on condition.
(C) A scenario in which one mutation (blue) changes the ancestral state (black) such that the average phenotype changes. A second mutation on the same haplotype (brown) changes expression such that the average phenotype is the same as the ancestral state. Thus the final phenotype averaged across the conditions is the same for both haplotypes, and the effects of the haplotype in the two conditions are in opposite directions.
(126 KB PDF)

Figure S6 The D30G Variant in *MKT1* Is Responsible for Transcript Variation Linking to the *MKT1* Region Segregant and replacement effects for distant *gxe*QTL with confidence intervals that overlap *MKT1* are shown. Each point represents a transcript. Solid lines indicate the best fit by linear regression (orange = BY, and purple = RM). The grey dotted line indicates *y* = *x*. Linear regression slopes, confidence intervals, and *R* ^2^ values are shown for each line. The slope is significantly different from 0, indicating that the variant contributes to phenotypic variation at this locus.
(187 KB PDF)

Table S1 Distant Peak Descriptions Each distant peak is described in further detail, with the location, number of linkages, verified or potential candidates, top significant GO term, and significant enriched transcription factor binding sites.
(29 KB XLS)

Table S2 Strain Descriptions A list of the strains used in this study.
(20 KB XLS)

Table S3 Parental Analysis Results A list of the results from the ANOVA for all transcripts tested. For each transcript, the presence of a polymorphism in the probe, *p*-values for each factor, percent variance explained for each factor, whether the effect was significant, and average effect size are listed.
(1.7 MB XLS)

Table S4 Linkage Analysis Results A list of all significant linkages. For each linkage, the presence of a polymorphism in the probe; the linkage peak marker, location, LOD score, confidence interval, and distant peak name; whether the peak is local or distant, and average effect sizes are listed.
(10.5 MB XLS)

Text S1 Discussion of Choice of Gene–Environment Interaction Analysis (44 KB DOC)

### Accession Numbers

Gene accession numbers reference the *Saccharomyces* Genome Database (http://www.yeastgenome.org): *AMN1* (S000000362), *CIN5* (S000005554, *GPA1* (S000001047), *HAP1* (S000004246), *HXT6* (S000002751) , *HXT7* (S000002750), *IDP2* (S000004164), *IRA2* (S000005441, *MATALPHA1* (S000000636), *MSN2* (S000004640),and *MSN4* (S000001545).

Expression raw data can be obtained via Gene Expression Omnibus (GEO; http://www.ncbi.nlm.nih.gov/projects/geo/) accession number GSE9376 and through PUMAdb (http://puma.princeton.edu/).

## Abbreviations

ANOVA: analysis of variance
CI: confidence interval
FDR: false discovery rate
GO: gene ontology
ORF: open reading frame
RR: relative risk

## References

[pbio-0060083-b001] Talmud PJ (2007). Gene-environment interaction and its impact on coronary heart disease risk. Nutr Metab Cardiovasc Dis.

[pbio-0060083-b002] Caspi A, Sugden K, Moffitt TE, Taylor A, Craig IW (2003). Influence of life stress on depression: moderation by a polymorphism in the 5-HTT gene. Science.

[pbio-0060083-b003] Ulrich CM, Kampman E, Bigler J, Schwartz SM, Chen C (1999). Colorectal adenomas and the C677T MTHFR polymorphism: evidence for gene-environment interaction. Cancer Epidemiol Biomarkers Prev.

[pbio-0060083-b004] Paterson AH, Damon S, Hewitt JD, Zamir D, Rabinowitch HD (1991). Mendelian factors underlying quantitative traits in tomato: comparison across species, generations, and environments. Genetics.

[pbio-0060083-b005] Ungerer MC, Halldorsdottir SS, Purugganan MD, Mackay TF (2003). Genotype-environment interactions at quantitative trait loci affecting inflorescence development in Arabidopsis thaliana. Genetics.

[pbio-0060083-b006] Li Y, Alvarez OA, Gutteling EW, Tijsterman M, Fu J (2006). Mapping determinants of gene expression plasticity by genetical genomics in C. elegans. PLoS Genet.

[pbio-0060083-b007] Rockman MV, Kruglyak L (2006). Genetics of global gene expression. Nat Rev Genet.

[pbio-0060083-b008] Gibson G, Weir B (2005). The quantitative genetics of transcription. Trends Genet.

[pbio-0060083-b009] Brem RB, Yvert G, Clinton R, Kruglyak L (2002). Genetic dissection of transcriptional regulation in budding yeast. Science.

[pbio-0060083-b010] Goring HH, Curran JE, Johnson MP, Dyer TD, Charlesworth J (2007). Discovery of expression QTLs using large-scale transcriptional profiling in human lymphocytes. Nat Genet.

[pbio-0060083-b011] Monks SA, Leonardson A, Zhu H, Cundiff P, Pietrusiak P (2004). Genetic inheritance of gene expression in human cell lines. Am J Hum Genet.

[pbio-0060083-b012] Morley M, Molony CM, Weber TM, Devlin JL, Ewens KG (2004). Genetic analysis of genome-wide variation in human gene expression. Nature.

[pbio-0060083-b013] Dixon AL, Liang L, Moffatt MF, Chen W, Heath S (2007). A genome-wide association study of global gene expression. Nat Genet.

[pbio-0060083-b014] Schadt EE, Monks SA, Drake TA, Lusis AJ, Che N (2003). Genetics of gene expression surveyed in maize, mouse and man. Nature.

[pbio-0060083-b015] Bystrykh L, Weersing E, Dontje B, Sutton S, Pletcher MT (2005). Uncovering regulatory pathways that affect hematopoietic stem cell function using ‘genetical genomics.'. Nat Genet.

[pbio-0060083-b016] Chesler EJ, Lu L, Shou S, Qu Y, Gu J (2005). Complex trait analysis of gene expression uncovers polygenic and pleiotropic networks that modulate nervous system function. Nat Genet.

[pbio-0060083-b017] Hubner N, Wallace CA, Zimdahl H, Petretto E, Schulz H (2005). Integrated transcriptional profiling and linkage analysis for identification of genes underlying disease. Nat Genet.

[pbio-0060083-b018] West MA, Kim K, Kliebenstein DJ, van Leeuwen H, Michelmore RW (2007). Global eQTL mapping reveals the complex genetic architecture of transcript-level variation in Arabidopsis. Genetics.

[pbio-0060083-b019] Brem RB, Storey JD, Whittle J, Kruglyak L (2005). Genetic interactions between polymorphisms that affect gene expression in yeast. Nature.

[pbio-0060083-b020] Storey JD, Akey JM, Kruglyak L (2005). Multiple locus linkage analysis of genomewide expression in yeast. PLoS Biol.

[pbio-0060083-b021] Brem RB, Kruglyak L (2005). The landscape of genetic complexity across 5,700 gene expression traits in yeast. Proc Natl Acad Sci U S A.

[pbio-0060083-b022] Landry CR, Oh J, Hartl DL, Cavalieri D (2006). Genome-wide scan reveals that genetic variation for transcriptional plasticity in yeast is biased towards multi-copy and dispensable genes. Gene.

[pbio-0060083-b023] Rolland F, Winderickx J, Thevelein JM (2002). Glucose-sensing and -signalling mechanisms in yeast. FEMS Yeast Res.

[pbio-0060083-b024] Brauer MJ, Saldanha AJ, Dolinski K, Botstein D (2005). Homeostatic adjustment and metabolic remodeling in glucose-limited yeast cultures. Mol Biol Cell.

[pbio-0060083-b025] DeRisi JL, Iyer VR, Brown PO (1997). Exploring the metabolic and genetic control of gene expression on a genomic scale. Science.

[pbio-0060083-b026] Kearney JF, Schutz MM, Boettcher PJ, Weigel KA (2004). Genotype x environment interaction for grazing versus confinement. I. Production traits. J Dairy Sci.

[pbio-0060083-b027] Hayes BJ, Carrick M, Bowman P, Goddard ME (2003). Genotype x environment interaction for milk production of daughters of Australian dairy sires from test-day records. J Dairy Sci.

[pbio-0060083-b028] Pigliucci M (2001). Phenotypic plasticity: beyond nature and nurture.

[pbio-0060083-b029] Falconer DS (1990). Selection in different environments: effects on environmental sensitivity (reaction norm) and on mean performance. Genet Res.

[pbio-0060083-b030] Ronald J, Brem RB, Whittle J, Kruglyak L (2005). Local regulatory variation in Saccharomyces cerevisiae. PLoS Genet.

[pbio-0060083-b031] GuhaThakurta D, Xie T, Anand M, Edwards SW, Li G (2006). Cis-regulatory variations: a study of SNPs around genes showing cis-linkage in segregating mouse populations. BMC Genomics.

[pbio-0060083-b032] Doss S, Schadt EE, Drake TA, Lusis AJ (2005). Cis-acting expression quantitative trait loci in mice. Genome Res.

[pbio-0060083-b033] Hovatta I, Zapala MA, Broide RS, Schadt EE, Libiger O (2007). DNA variation and brain region-specific expression profiles exhibit different relationships between inbred mouse strains: implications for eQTL mapping studies. Genome Biol.

[pbio-0060083-b034] Stranger BE, Nica AC, Forrest MS, Dimas A, Bird CP (2007). Population genomics of human gene expression. Nat Genet.

[pbio-0060083-b035] Yvert G, Brem RB, Whittle J, Akey JM, Foss E (2003). Trans-acting regulatory variation in Saccharomyces cerevisiae and the role of transcription factors. Nat Genet.

[pbio-0060083-b036] Miyajima I, Nakafuku M, Nakayama N, Brenner C, Miyajima A (1987). GPA1, a haploid-specific essential gene, encodes a yeast homolog of mammalian G protein which may be involved in mating factor signal transduction. Cell.

[pbio-0060083-b037] Dietzel C, Kurjan J (1987). The yeast SCG1 gene: a G alpha-like protein implicated in the a- and alpha-factor response pathway. Cell.

[pbio-0060083-b038] Tedford K, Kim S, Sa D, Stevens K, Tyers M (1997). Regulation of the mating pheromone and invasive growth responses in yeast by two MAP kinase substrates. Curr Biol.

[pbio-0060083-b039] Bardwell L, Cook JG, Zhu-Shimoni JX, Voora D, Thorner J (1998). Differential regulation of transcription: repression by unactivated mitogen-activated protein kinase Kss1 requires the Dig1 and Dig2 proteins. Proc Natl Acad Sci U S A.

[pbio-0060083-b040] MacIsaac KD, Wang T, Gordon DB, Gifford DK, Stormo GD (2006). An improved map of conserved regulatory sites for Saccharomyces cerevisiae. BMC Bioinformatics.

[pbio-0060083-b041] Harbison CT, Gordon DB, Lee TI, Rinaldi NJ, Macisaac KD (2004). Transcriptional regulatory code of a eukaryotic genome. Nature.

[pbio-0060083-b042] Storey JD, Tibshirani R (2003). Statistical significance for genomewide studies. Proc Natl Acad Sci U S A.

[pbio-0060083-b043] Broach JR (1991). Ras-regulated signaling processes in Saccharomyces cerevisiae. Curr Opin Genet Dev.

[pbio-0060083-b044] Buchberg AM, Cleveland LS, Jenkins NA, Copeland NG (1990). Sequence homology shared by neurofibromatosis type-1 gene and IRA-1 and IRA-2 negative regulators of the RAS cyclic AMP pathway. Nature.

[pbio-0060083-b045] Landry CR, Lemos B, Rifkin SA, Dickinson WJ, Hartl DL (2007). Genetic properties influencing the evolvability of gene expression. Science.

[pbio-0060083-b046] Deutschbauer AM, Davis RW (2005). Quantitative trait loci mapped to single-nucleotide resolution in yeast. Nat Genet.

[pbio-0060083-b047] Ben-Ari G, Zenvirth D, Sherman A, David L, Klutstein M (2006). Four linked genes participate in controlling sporulation efficiency in budding yeast. PLoS Genet.

[pbio-0060083-b048] Sinha H, Nicholson BP, Steinmetz LM, McCusker JH (2006). Complex genetic interactions in a quantitative trait locus. PLoS Genet.

[pbio-0060083-b049] Lynch M, Walsh B (1998). Genetics and analysis of quantitative traits.

[pbio-0060083-b050] Broman KW, Wu H, Sen S, Churchill GA (2003). R/qtl: QTL mapping in experimental crosses. Bioinformatics.

[pbio-0060083-b051] Kruglyak L, Lander ES (1995). A nonparametric approach for mapping quantitative trait loci. Genetics.

[pbio-0060083-b052] Dupuis J, Siegmund D (1999). Statistical methods for mapping quantitative trait loci from a dense set of markers. Genetics.

[pbio-0060083-b053] Gray M, Piccirillo S, Honigberg SM (2005). Two-step method for constructing unmarked insertions, deletions and allele substitutions in the yeast genome. FEMS Microbiol Lett.

[pbio-0060083-b054] Brachmann CB, Davies A, Cost GJ, Caputo E, Li J (1998). Designer deletion strains derived from Saccharomyces cerevisiae S288C: a useful set of strains and plasmids for PCR-mediated gene disruption and other applications. Yeast.

[pbio-0060083-b055] Sealfon RS, Hibbs MA, Huttenhower C, Myers CL, Troyanskaya OG (2006). GOLEM: an interactive graph-based gene-ontology navigation and analysis tool. BMC Bioinformatics.

[pbio-0060083-b056] McDonald JH, Kreitman M (1991). Adaptive protein evolution at the Adh locus in Drosophila. Nature.

[pbio-0060083-b057] Thornton K (2003). Libsequence: a C++ class library for evolutionary genetic analysis. Bioinformatics.

[pbio-0060083-b058] Wei W, McCusker JH, Hyman RW, Jones T, Ning Y (2007). Genome sequencing and comparative analysis of Saccharomyces cerevisiae strain YJM789. Proc Natl Acad Sci U S A.

[pbio-0060083-b059] Kellis M, Patterson N, Endrizzi M, Birren B, Lander ES (2003). Sequencing and comparison of yeast species to identify genes and regulatory elements. Nature.

[pbio-0060083-b060] Pond SL, Frost SD, Muse SV (2005). HyPhy: hypothesis testing using phylogenies. Bioinformatics.

