# Supplementary material for: Gene–Environment Interaction in Yeast Gene Expression
Source: PLoS Biol. 2008 Apr 15;6(4):e83. doi: 10.1371/journal.pbio.0060083 (PMC2292755; doi:10.1371/journal.pbio.0060083)
Supplement: Supplementary file 1 [file pbio.0060083.sg001.pdf]

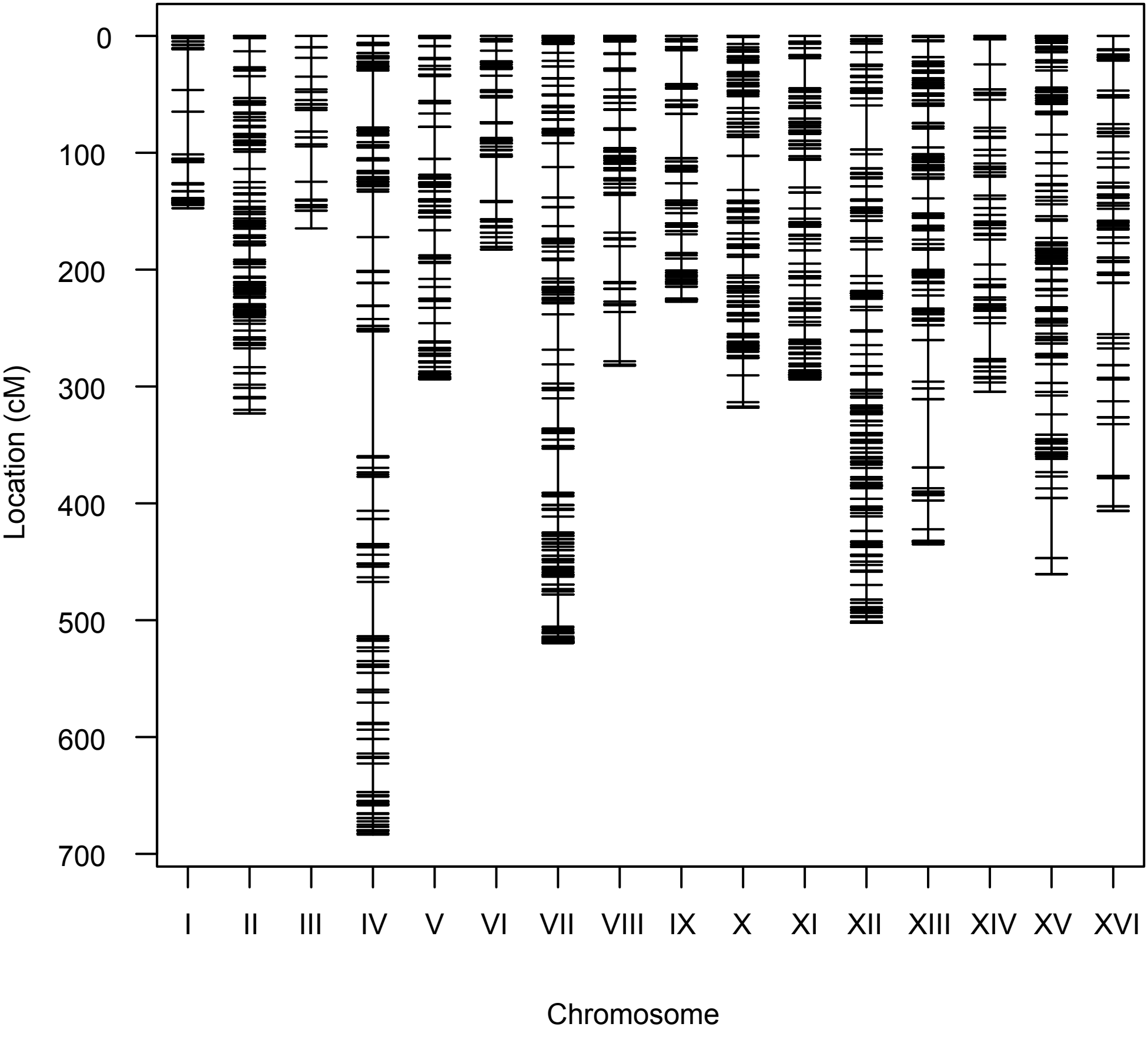

0
100
200
300
400
500
600
700
Location (cM)
I
II
III
IV
V
VI
VII
VIII
IX
X
XI
XII
XIII
XIV
XV
XVI
Chromosome
