# Supplementary material for: Gene–Environment Interaction in Yeast Gene Expression
Source: PLoS Biol. 2008 Apr 15;6(4):e83. doi: 10.1371/journal.pbio.0060083 (PMC2292755; doi:10.1371/journal.pbio.0060083)
Supplement: Supplementary file 2 [file pbio.0060083.sg002.pdf]

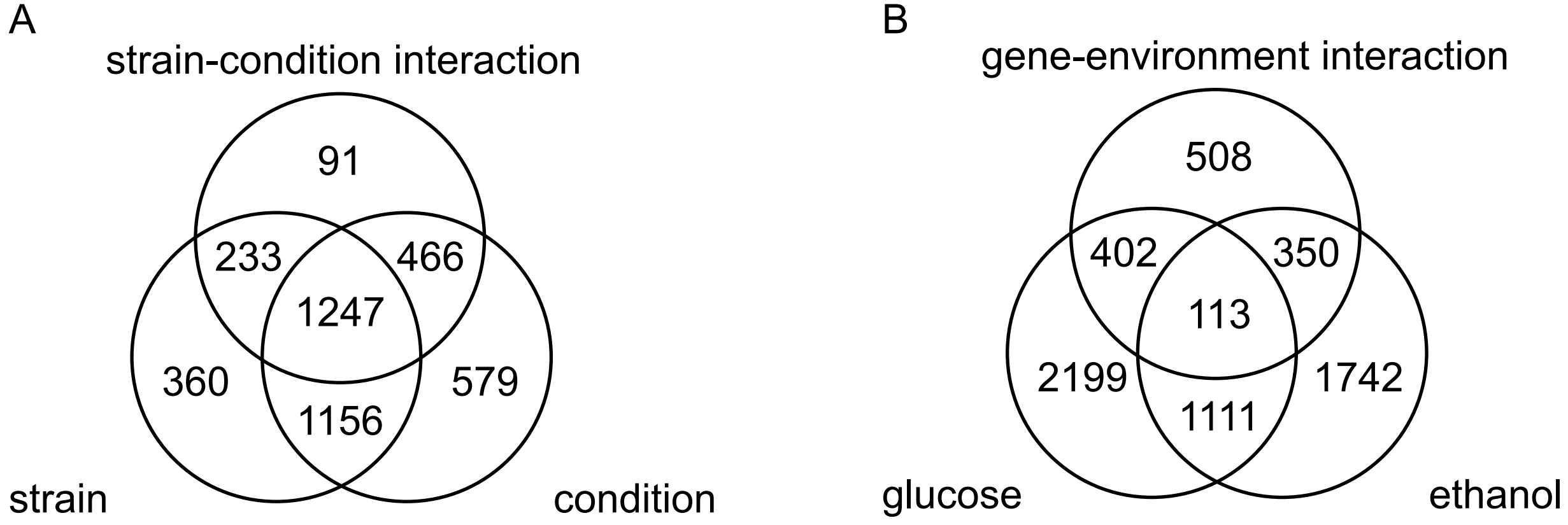
A
strain-condition interaction
91
233
466
1247
360
579
1156
strain
condition
B
gene-environment interaction
508
402
350
113
2199
1742
1111
glucose
ethanol
