# Supplementary material for: Gene–Environment Interaction in Yeast Gene Expression
Source: PLoS Biol. 2008 Apr 15;6(4):e83. doi: 10.1371/journal.pbio.0060083 (PMC2292755; doi:10.1371/journal.pbio.0060083)
Supplement: Supplementary file 3 [file pbio.0060083.sg003.pdf]

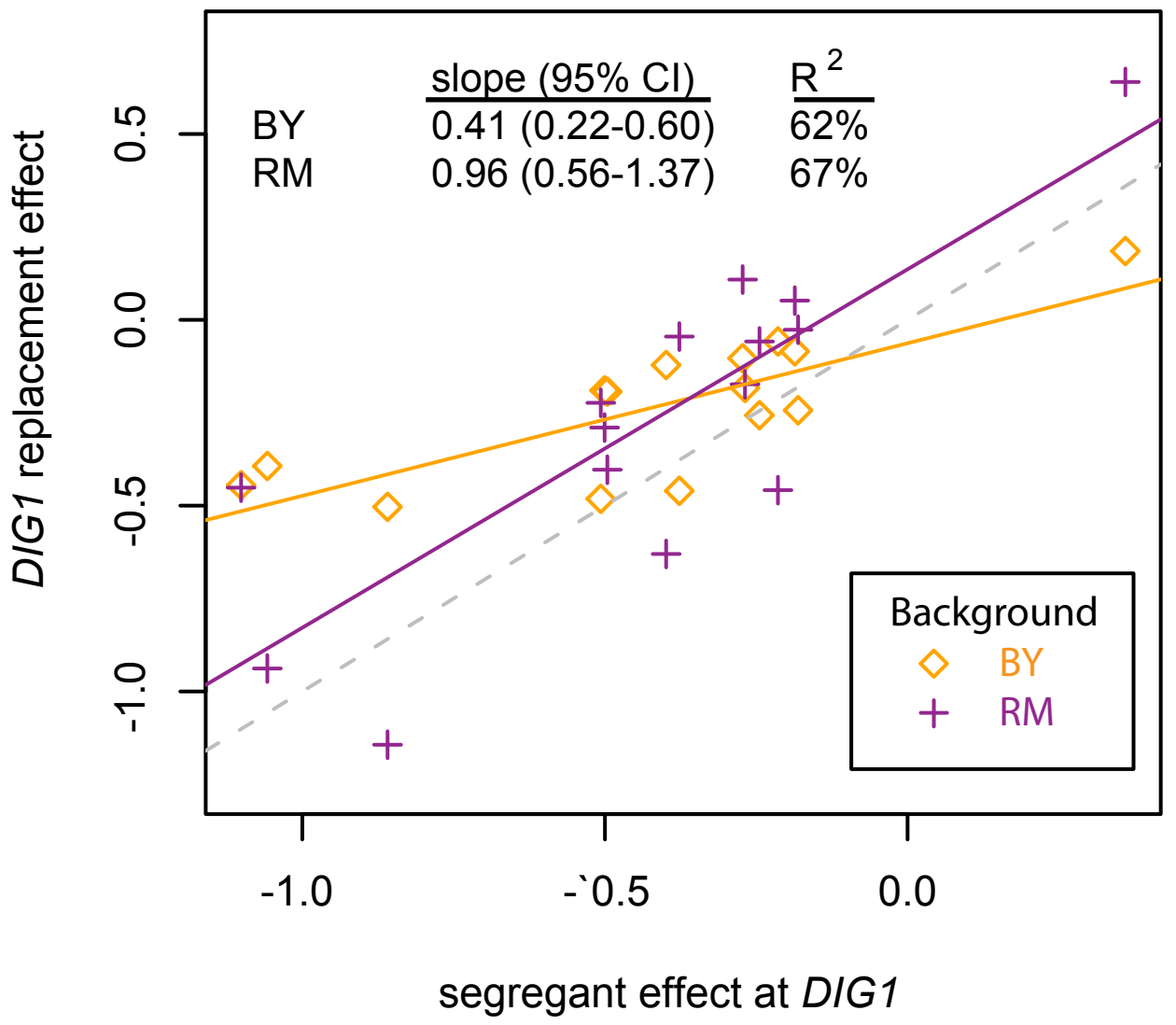
slope (95% CI)
R 2
BY
0.41 (0.22-0.60)
62%
RM
0.96 (0.56-1.37)
67%
Background
BY
RM
0.5
0.0
-0.5
-1.0
-1.0
-`0.5
0.0
DIG1 replacement effect
segregant effect at *DIG1*
