# Supplementary material for: Gene–Environment Interaction in Yeast Gene Expression
Source: PLoS Biol. 2008 Apr 15;6(4):e83. doi: 10.1371/journal.pbio.0060083 (PMC2292755; doi:10.1371/journal.pbio.0060083)
Supplement: Supplementary file 4 [file pbio.0060083.sg004.pdf]

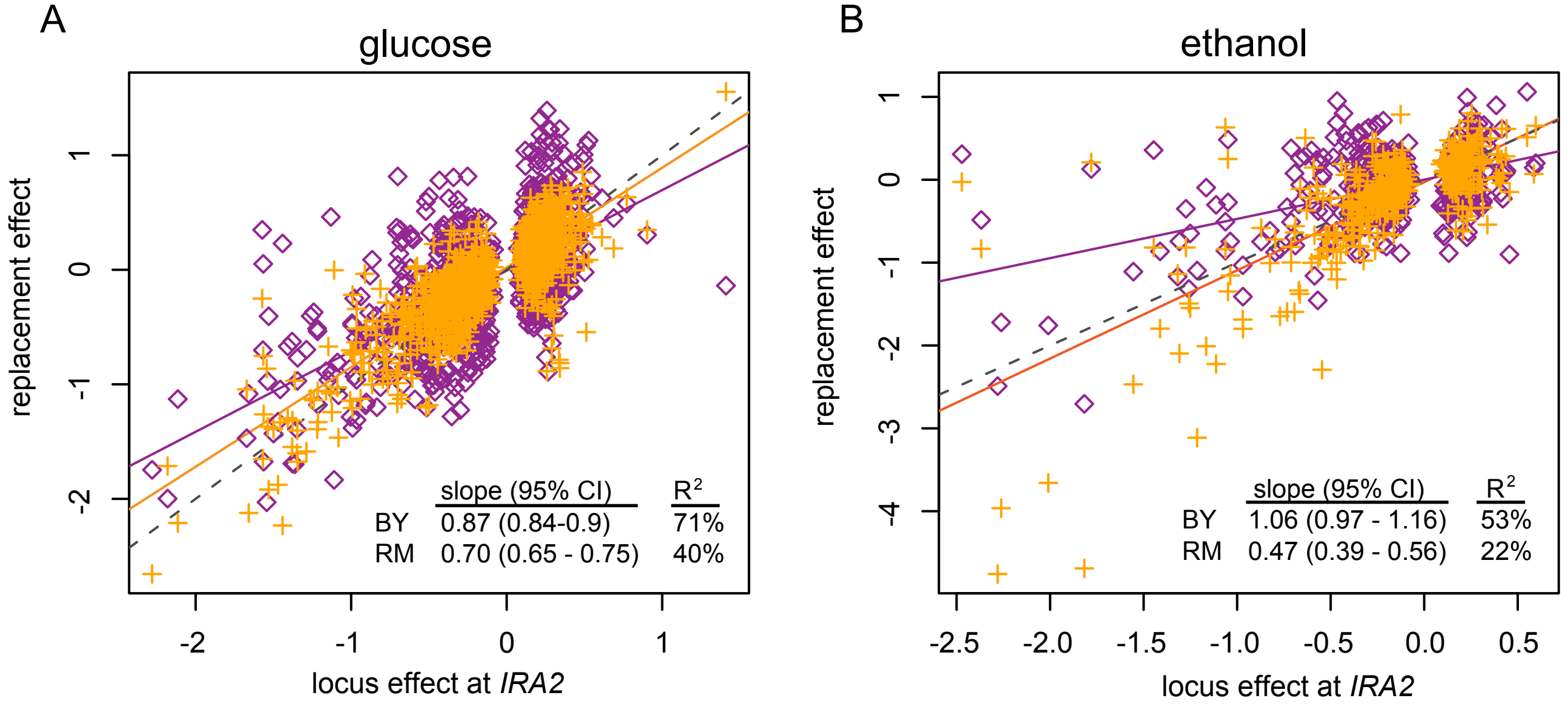

A
glucose
replacement effect
locus effect at IRA2
slope (95% CI)
R^2
BY 0.87 (0.84-0.9) 71%
RM 0.70 (0.65 - 0.75) 40%
B
ethanol
replacement effect
locus effect at IRA2
slope (95% CI)
R^2
BY 1.06 (0.97 - 1.16) 53%
RM 0.47 (0.39 - 0.56) 22%
