# Supplementary material for: Gene–Environment Interaction in Yeast Gene Expression
Source: PLoS Biol. 2008 Apr 15;6(4):e83. doi: 10.1371/journal.pbio.0060083 (PMC2292755; doi:10.1371/journal.pbio.0060083)
Supplement: Supplementary file 5 [file pbio.0060083.sg005.pdf]

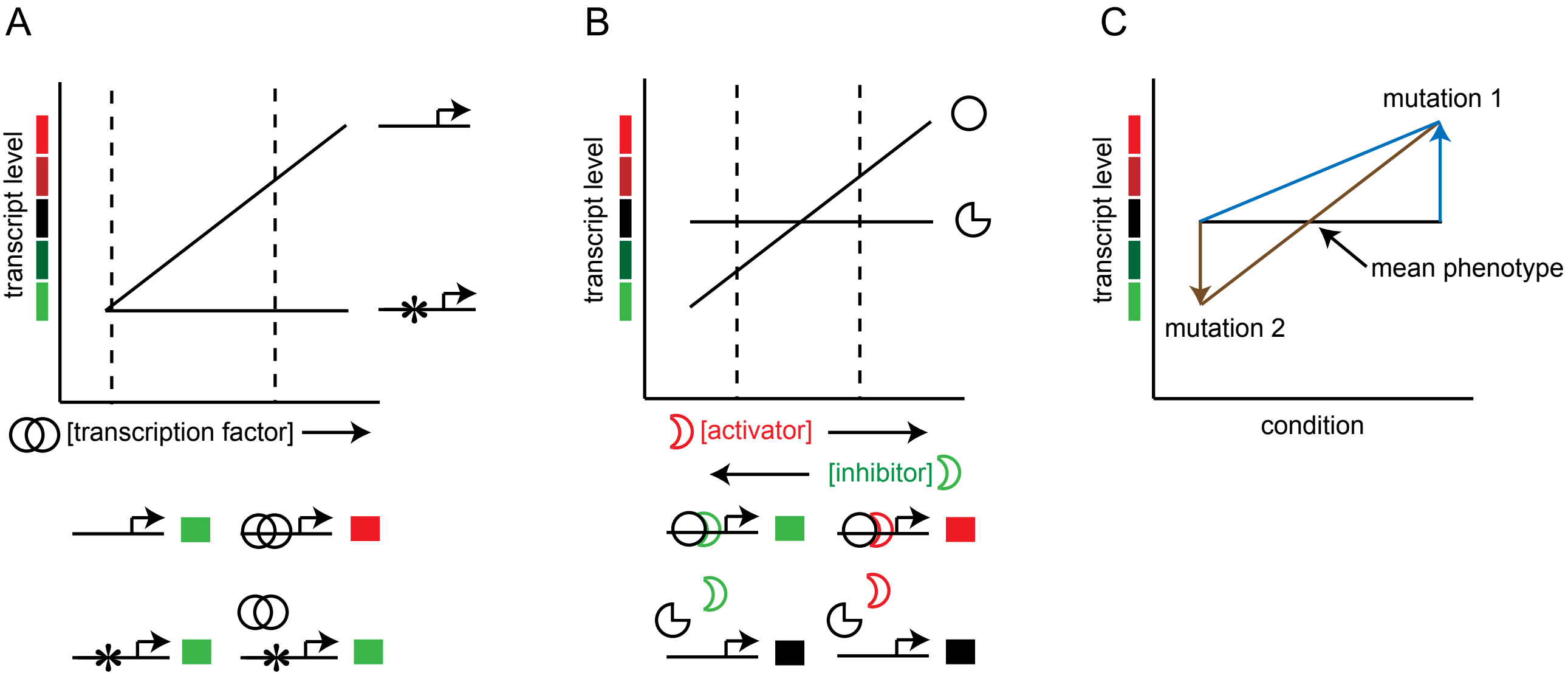
A
transcript level
[transcription factor]
B
transcript level
[activator]
[inhibitor]
C
transcript level
mutation 1
mean phenotype
mutation 2
condition
