# Supplementary material for: Gene–Environment Interaction in Yeast Gene Expression
Source: PLoS Biol. 2008 Apr 15;6(4):e83. doi: 10.1371/journal.pbio.0060083 (PMC2292755; doi:10.1371/journal.pbio.0060083)
Supplement: Supplementary file 6 [file pbio.0060083.sg006.pdf]

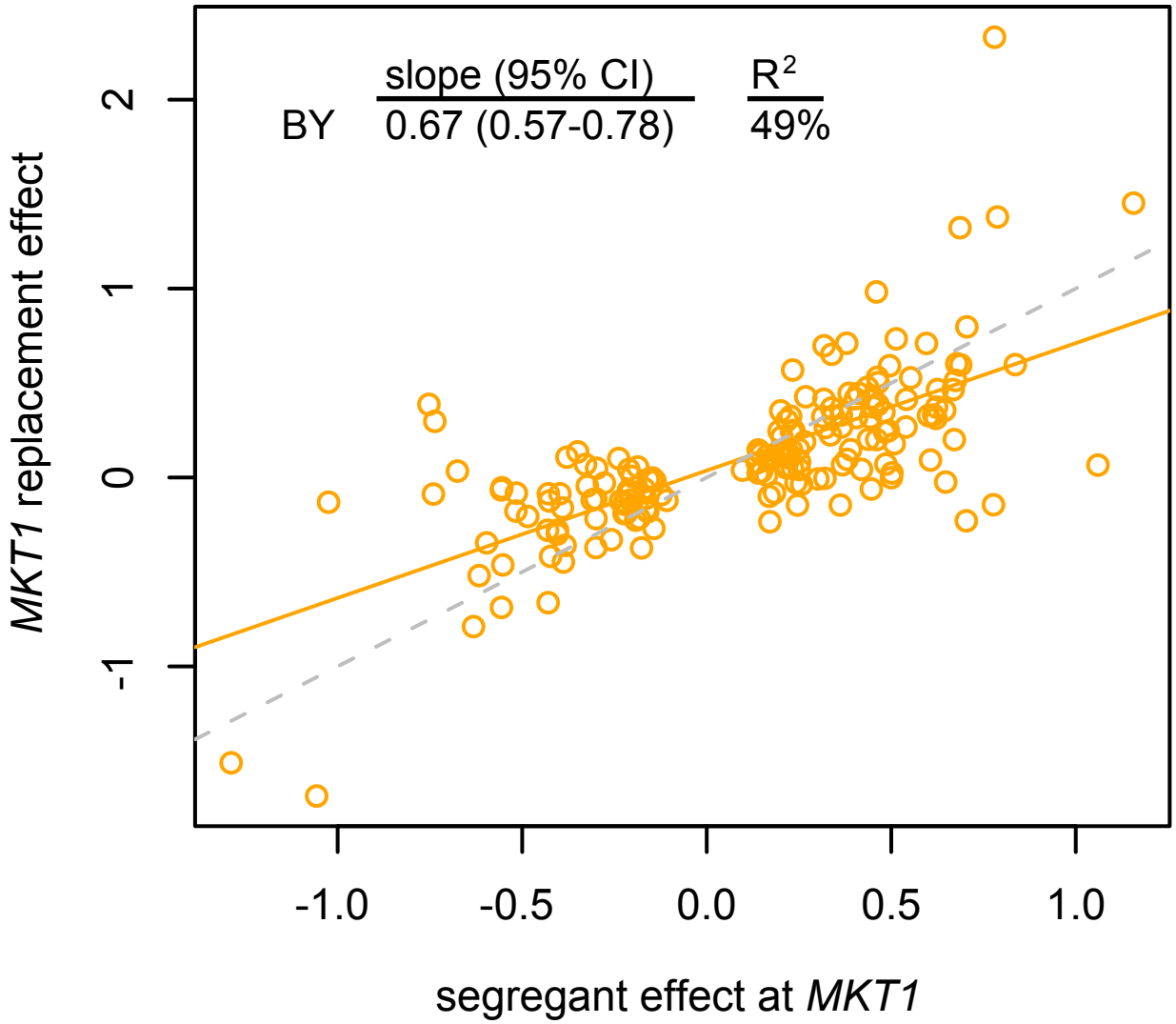
slope (95% CI)
R2
BY
0.67 (0.57-0.78)
49%
MKT1 replacement effect
segregant effect at MKT1
-1.0
-0.5
0.0
0.5
1.0
-1
0
1
2
