# Supplementary material for: Gene–Environment Interaction in Yeast Gene Expression
Source: PLoS Biol. 2008 Apr 15;6(4):e83. doi: 10.1371/journal.pbio.0060083 (PMC2292755; doi:10.1371/journal.pbio.0060083)
Supplement: Supplementary file 7 [file pbio.0060083.sd003.doc]

**Assessing gene-environment interaction**

Gene-environment interaction can be quantified in a number of ways, and the choice of measurement is dependent on study design. When the same strain is measured in multiple conditions, such that the measurements are paired, one can use the environmental sensitivity approach. Environmental sensitivity is a measure of change between conditions and is defined as the difference between trait values in two conditions, standardized by the average environmental effect [1]. The standardization term is the same for all strains, so when comparing strains (as in linkage analysis), this term is not needed, and the sensitivity can be reduced to simply the difference between conditions. When a locus is associated with environmental sensitivity, then that locus is considered to show gene-environment interaction. A major advantage of this measurement is that all loci can rapidly be tested for interaction, without being restricted to loci that are significant in at least one condition. Due to its simplicity of measurement, easy adaptability to existing linkage analysis software, and the biological relevance of a phenotype that indicates how much individuals respond to their environment, we chose to use this method for linkage analysis.

When multiple replicates are present of the same strain in different conditions, one can use Analysis of Variance (ANOVA) [2]. After correcting for average strain and average condition effects, the interaction term arises from the variance that can be explained by considering strain and condition effects jointly. ANOVA is well suited for estimating gene-environment interactions in unrelated strains, and we use it here to estimate interactions present between our two parental strains, BY and RM. It is a common approach to use ANOVA to test individual loci for interactions after identifying them in genome-wide scans. We chose not to use this method because we would be restricted to loci that are genome-wide significant in at least one condition which would miss many *gxe*QTL (see main text). Additionally, we felt that ANOVA does not adequately take into account the paired nature of the experiment: the same segregants are measured in the two conditions, and the environmental sensitivity analysis (which takes advantage of this pairing) is expected to be more powerful than an unpaired ANOVA.

Interaction can also be characterized by treating the phenotypes in two conditions as genetically correlated traits [3]. The two traits can be strongly positively correlated if there is no interaction or if there are scale effects such that one strain always has a phenotype larger than the other, but to a different extent in the two conditions. The traits would be strongly negatively correlated if the reaction norms crossed, such that one strain was higher in one condition, but lower in the other [1]. A trait with a genetic correlation significantly less than 1 is expected to show gene-environment interaction by ANOVA [1]. Multi-trait composite interval mapping builds on this framework to jointly map multiple traits simultaneously [4]. For a paired study design, gene-environment interaction is assessed in two steps. First, the two phenotypes are mapped jointly, allowing for detection of loci that are significant in at least one condition. By mapping both phenotypes jointly, one gains power to detect loci when the traits have a genetic correlation greater than 0. The second step assesses gene-environment interaction by asking whether the additive effects (and/or dominance effects in a diploid) are the same in both conditions. This is mathematically equivalent to asking whether the effect of the condition is the same for both alleles, which is the approach taken by the environmental sensitivity method we use. Thus, the difference between the two tests is primarily a difference in which loci get tested for interaction. In the environmental sensitivity method, all loci are tested, while in multi-trait composite interval mapping, only loci that are significant in at least one condition are tested (which as noted before would miss many *gxe*QTL). In addition, multi-trait composite interval mapping is a parametric procedure that makes strong assumptions about the phenotypic distribution. Variation in environmental sensitivity, however, can be mapped directly using nonparametric tests that retain statistical validity and power even when these assumptions are violated [5].

1. Falconer DS (1990) Selection in Different Environments - Effects on Environmental Sensitivity (Reaction Norm) and on Mean Performance. Genetical Research 56: 57-70.

2. Sokal RR, Rohlf FJ (1994) Biometry : the principles and practice of statistics in biological research. New York: Freeman. xix, 887 p.

3. Falconer DS (1952) The Problem of Environment and Selection. American Naturalist 86: 293-298.

4. Jiang C, Zeng ZB (1995) Multiple trait analysis of genetic mapping for quantitative trait loci. Genetics 140: 1111-1127.

5. Kruglyak L, Lander ES (1995) A nonparametric approach for mapping quantitative trait loci. Genetics 139: 1421-1428.
